# Sorting of SEC translocase SCY components to different membranes in chloroplasts

**DOI:** 10.1093/jxb/erx318

**Published:** 2017-09-13

**Authors:** Rajneesh Singhal, Donna E Fernandez

**Affiliations:** Department of Botany, University of Wisconsin-Madison, Madison, WI, USA

**Keywords:** SEC translocases, chloroplast SRP pathway, inner envelope, protein targeting, SCY1, SCY2, SRP43, SRP54, thylakoids

## Abstract

Membrane proteins that are imported into chloroplasts must be accurately routed in order to establish and maintain the highly differentiated membranes characteristic of these organelles. Little is known about the targeting information or pathways involved, especially in the case of proteins with multiple transmembrane domains. We have studied targeting of the SCY components of the two SEC translocases in chloroplasts. SCY1 and SCY2 share a similar, highly conserved structure with 10 transmembrane domains, but are targeted to different membranes: the thylakoids and inner envelope, respectively. We used protoplast transfections and a confocal microscopy imaging assay in combination with a domain-swapping approach to investigate sorting pathways and identify important targeting elements in these proteins. We show that the N-terminal region of SCY1 contains targeting determinants that allow SCY1 to be recruited to the signal-recognition particle pathway. In addition, substituting the N-terminal region of SCY1 for the N-terminal region of SCY2 causes SCY2 to be displaced out of the inner envelope. The region of SCY2 that contains transmembrane domains 3 and 4 is necessary for localization to the inner envelope and may serve as a membrane anchor, enhancing the integration of other transmembrane domains via either stop-transfer or post-import mechanisms.

## Teaser

Targeting determinants in the N-terminal region direct SCY1 to the thylakoids via the signal-recognition particle pathway, while specific transmembrane domains are needed to localize SCY2 in the inner envelope.

## Introduction

Protein import and targeting within the chloroplast involves both novel and ancient translocases present in its membranes (Celedon and Cline, 2013; Zimorski *et al.*, 2014). Approximately 3000 proteins encoded in the nuclear genome are translated on cytosolic ribosomes and need to be targeted to one of six different sub-compartments, which include three membranes (outer envelope, inner envelope, and thylakoid), and three aqueous compartments (intermembrane space, stroma, and thylakoid lumen) (Leister, 2003; López-Juez, 2007). Most imported chloroplast proteins are synthesized as pre-proteins with an N-terminal extension called a transit peptide, which guides the protein to the surface of the chloroplast (Li and Teng, 2013; Shi and Theg, 2013; Paila *et al.*, 2015). Pre-proteins are recognized by the receptor components of the Translocon at the Outer envelope membrane of Chloroplasts (TOC) complex and directed through the channel formed by TOC75 to the Translocon at the Inner envelope membrane of Chloroplasts (TIC) complex (Nakai, 2015; Paila *et al.*, 2015; Sjuts *et al.*, 2017). As soon as the transit peptide emerges from the TIC channel, it is removed by the stromal processing peptidase (Richter and Lamppa, 1998; Trösch and Jarvis, 2011). Further import is driven by molecular chaperones such as heat-shock protein (HSP) 70/HSP93, which act as import motors. Upon entering the stroma, the imported protein either assumes its functional conformation (stromal proteins) or is trafficked further (inner envelope, thylakoid membrane, and thylakoid lumen proteins) (Huang *et al.*, 2016; Sjuts *et al.*, 2017). Thylakoid proteins are directed to translocase/integrase systems that are homologous to bacterial systems and include the thylakoid SEC1, twin arginine translocase (TAT), and signal-recognition particle (SRP)/ALBINO3 (ALB3) systems (Schünemann, 2007; Celedon and Cline, 2013; Ziehe *et al.*, 2017).

How imported membrane proteins are sorted to either the inner envelope or thylakoids is still not properly understood. Proteins destined for the inner envelope are thought to follow two main pathways. Some inner-envelope proteins appear to undergo translocation arrest at the inner envelope membrane and are released from the TIC translocon into the lipid bilayer. This is known as the stop-transfer pathway, and is used by several single transmembrane domain (TMD) proteins with N-termini oriented toward the stroma, such as accumulation and replication of chloroplasts 6 (ARC6) and albino or pale green mutant 1 (APG1) (Motohashi *et al.*, 2003; Viana *et al.*, 2010; Froehlich and Keegstra, 2011). Because the protein never leaves the plane of the bilayer, soluble stromal intermediates cannot be detected. The TMD appears to serve as the inner-envelope targeting determinant in these cases. When the TMD of ARC6 was replaced by TMDs from thylakoid membrane proteins, the chimeric proteins were directed to the thylakoids. Conversely, when the TMD of the thylakoid membrane protein plastidic type 1 signal peptidase 1 (PLSP1) was replaced with that of ARC6, the chimeric protein associated with the inner envelope (Froehlich and Keegstra, 2011). By contrast, at least three inner-envelope proteins completely cross the inner membrane and insert into the bilayer from the stromal side. During import reactions, three components of the TIC (TIC40, TIC110, and TIC21) appear briefly as soluble intermediates in the stroma (Lübeck *et al.*, 1997; Li and Schnell, 2006; Teng *et al.*, 2006; Tripp *et al.*, 2007; Firlej-Kwoka *et al.*, 2008), which indicates they use a post-import or conservative sorting pathway. For TIC40, the single TMD near the N-terminus, combined with an adjacent Ser/Pro-rich domain, was shown to be necessary and sufficient for integration (Tripp *et al.*, 2007). Recently published work has shown that the inner envelope-localized SEC2 system mediates integration of TIC40 (Li *et al.*, 2017). For other inner-envelope membrane proteins where soluble intermediates have not been identified, such as FTSH12, the TMD may be integrated from the stroma side by SEC2 while the rest of the protein is still transiting the TIC channel.

Having multiple TMDs adds another layer of complexity and relatively few studies have addressed the targeting of polytopic membrane proteins within chloroplasts. In the most well-studied case, that of the highly abundant light-harvesting chlorophyll binding proteins (LHCPs), which have three TMDs, a small amino acid motif (DPLG) within the L18 domain of the mature proteins facilitates recognition by components of the chloroplast SRP pathway (DeLille *et al.*, 2000). The SRP pathway utilizes two proteins when operating in a post-translational mode: SRP54, which is a homolog of the bacterial SRP54 protein, and SRP43, which is a chloroplast-specific chaperone (Schünemann, 2007). Binding by SRP43, either alone or as a heterodimer with SRP54, results in delivery of LHCPs to receptors in the thylakoid membrane (Kogata *et al.*, 1999; Tu *et al.*, 1999). The delivered proteins are integrated into the thylakoid membrane by ALB3, a member of the ALB3/YidC/Oxa1 family of translocase/integrase proteins (Moore *et al.*, 2000). Studies of the targeting of polytopic proteins to the inner envelope have identified one or more TMDs as being important (Knight and Gray, 1995; Teng *et al.*, 2006; Sattasuk *et al.*, 2011; Okawa *et al.*, 2014), but we lack a clear picture of the pathways and machinery involved in integration.

To further our understanding of membrane-specific sorting in chloroplasts, we have studied the targeting behavior of components of the chloroplast SEC translocases. The SEC1 translocase is restricted to the thylakoids, while the SEC2 translocase is found in the inner envelope (Skalitzky *et al.*, 2011). Despite these differences in localization, the membrane components have highly conserved structures. Therefore, unlike experiments that involve swapping domains between unrelated proteins, the overall topology and relative position of the domains can be maintained in domain-swapping experiments. In this work, we focused primarily on the SCY components, which are homologous to bacterial SecY proteins and have 10 TMDs that form the translocation channel (Supplementary Fig. S1 at *JXB* online). We used an imaging assay based on localization of fluorescent fusion proteins in transfected protoplasts by laser confocal microscopy, which provides good spatial resolution and allows for higher throughput than biochemical methods that involve fractionation of chloroplasts. We generated and analysed a large number of different combinations of domains but focus in this report on experiments involving the N-terminal region and first few TMDs, which carry elements that appear to be particularly important for high-fidelity targeting of these proteins. Our experiments show that the N-terminal region of SCY1 contains targeting determinants that allow SCY1 to be recruited to the SRP pathway and that are sufficient to displace the 10 TMDs of SCY2 out of the inner envelope. The region of SCY2 that contains TMD3 and TMD4 is necessary for localization to the inner envelope and may serve as a membrane anchor, enhancing the integration of other TMDs via either stop-transfer or post-import mechanisms.

## Materials and methods

### Plant material and growth conditions

*Arabidopsis thaliana* (L.) seeds were surface-sterilized, stratified at 4 °C for 48 h, and grown on 0.8% agar plates containing half-strength Murashige and Skoog (MS) media with vitamins (Caisson Labs), 50 mM MES/KOH pH 5.7, 1% w/v sucrose, in a growth chamber at 22 °C with a 12/12 h light/ dark light cycle. After 7 d, the seedlings were transferred to soil and maintained under the same growth conditions.

Seed stocks for plants containing mutations in *SRP43* (AT2G47450) and *SRP54* (AT5G03940) were obtained from the Arabidopsis Biological Resource Center (*srp 43*: SAIL_783_F08, *srp54*: WiscDsLox289_292B14; https://abrc.osu.edu/). DNA was isolated from the leaves of individual plants as described previously (Adamczyk *et al.*, 2007). Plants carrying mutant alleles were identified by PCR-genotyping with gene-specific and T-DNA-specific primers, shown in Supplementary Table S1. The *srp43* allele had an insertion near the 3′-end of the coding sequence, and was presumed to be a loss-of-function allele based on the decrease in chlorophyll levels in homozygous mutant plants. The *srp54* allele had an insertion in the seventh exon, and was previously characterized as a loss-of-function allele (Yu *et al.*, 2012).

### Generation of GFP-fusion constructs and transmembrane domain analysis

Primers used in the generation of green fluorescent protein (GFP) fusion constructs are listed in Supplementary Table S1. Total RNA was isolated from 2-week-old wild-type Arabidopsis (Columbia ecotype) plants using Trizol reagent (Thermo Fisher Scientific) according to the manufacturer’s instructions. cDNA was synthesized using 1 µg of total RNA, as described previously (Lehti-Shiu *et al.*, 2005), and used as a template to PCR-amplify full-length SCY1 (AT2G18710) and SCY2 (AT2G31530) coding sequences minus the stop codon. The PCR fragments were cloned into the pML94 vector (Bionda *et al.*, 2010) between the KpnI and SpeI restriction sites by sequence- and ligation-independent cloning (SLIC) (Jeong *et al.*, 2012), creating an in-frame fusion with a c-myc linker and GFP coding sequence. The vector also included the 35S promoter and a NOS terminator. All other SCY1–SCY2 chimeric constructs were generated using primers that spanned the SCY1–SCY2 fusion junctions and overlap-extension PCR, followed by SLIC. To predict the TMDs, Clustal Omega (Sievers *et al.*, 2011) was used to align SCY1, SCY2, and *E. coli* SecY sequences and regions were identified that correspond to the *E. coli* TMDs, as determined by cryo-electron microscopy (Park *et al.*, 2014). For truncated proteins, the SCY sequence was terminated either in the loop region between the TMDs or directly after the TMD if the loop between the TMDs was very short. The Arabidopsis TIC20-mCherry construct was created by modifying a TIC20-GFP construct in pML94 (Machettira *et al.*, 2012), obtained from Prof. Enrico Schleiff (Department of Biosciences, Goethe University, Germany). All constructs used for imaging were verified by sequencing. For each predicted SCY1 and SCY2 TMD, the mean hydrophobicity of residues was calculated based on their octanol/water partition coefficients using the HELIQUEST web server (http://heliquest.ipmc.cnrs.fr) and a full amino acid window (Gautier *et al.*, 2008). The relative helical propensity was assessed by calculating the Agadir score at pH7.5, 298 K, ionic strength 0.15 M, using the Agadir calculator (http://agadir.crg.es), which predicts the helical behavior of monomeric peptides in aqueous solutions using an algorithm based on helix/coil transition theory (Muñoz and Serrano, 1997).

### Protoplast isolation and transfection

Protoplast isolation and transfection was carried out as described previously (Wu *et al.*, 2009). Ten to twelve large leaves were removed from 3-week-old wild-type (Ws ecotype) Arabidopsis plants, and the lower epidermis was peeled off using Scotch^®^ Magic™ tape (3M) while the leaf was supported by Fisherbrand™ label tape adhered to the upper epidermis. The leaves were then transferred to a petri dish containing 10 ml of digestive solution (1% cellulase ‘Onozuka’ R10, 0.25% macerozyme ‘Onozuka’ R10, 0.4 M mannitol, 10 mM CaCl_2_, 20 mM KCl, 0.1% BSA and 20 mM MES pH5.7) and incubated with gentle shaking on a platform shaker for 1 h. The released protoplasts were then centrifuged at 120 *g* for 5 min. The protoplasts were washed once with 10 ml of W5 solution (154 mM NaCl, 125 mM CaCl_2_, 5 mM KCl, 5 mM glucose, and 2 mM MES pH 5.7), resuspended again in W5 solution, and incubated for 30 min on ice followed by centrifugation at 120 *g* for 5 min. The collected protoplasts were counted using a haemocytometer and light microscope and resuspended in MMG solution (0.4 M mannitol, 15 mM MgCl_2_, and 4 mM MES pH 5.7) to a final concentration of 1 × 10^6^ cells ml^–1^. For transfection, approximately 100 000 protoplasts in 100 μl were mixed with 10 μg of plasmid DNA in a 2-ml Eppendorf tube. An equal volume of 40% PEG solution (40% w/v, PEG MW 4000, 0.1 M CaCl_2_, and 0.2 M mannitol) was added and the mixture was incubated at room temperature for 5 min. After incubation, 1.5 ml of W5 solution was added slowly and the protoplasts were collected by centrifuging at 82 *g* for 2 min in a table-top microcentrifuge. The protoplasts were then resuspended in 1 ml W5 solution and incubated in 6-well plates overnight under dark or light conditions for 12–16 h. The protoplasts were transferred to 18-well flat-bottomed µ-Slides (http://ibidi.com) for microscopy. For experiments that involved overnight exposure to light, protoplasts were isolated from plants grown on a reverse 12 h night/12 h day cycle. After isolating and transfecting the protoplasts, the protoplasts were incubated in the growth chamber with the lights on for the 12 h corresponding to their day cycle. This ensured that the circadian rhythm was maintained and resulted in a higher frequency of healthy protoplasts.

### Microscopy and data analysis

The protoplast images in each figure are representative of at least three independent experiments. In each experiment, at least 7–10 transfected protoplasts were examined. For constructs V and VI, transfected protoplasts were imaged using a 40×/1.2 W C-Apochromat lens and LSM 510 Meta confocal laser scanning microscope (Zeiss, http://www.zeiss.com/microscopy/us). GFP and chlorophyll were excited using the 488-nm laser, and emission was recorded at 500–530 nm and 650–700 nm respectively, with the images captured in channel mode. For the remaining constructs, transfected protoplasts were imaged using a 40×/1.1 W C-Apochromat lens and inverted LSM 780 confocal laser scanning microscope (Zeiss). GFP and chlorophyll were excited using the 488-nm laser, and the emission spectrum was recorded in lambda mode in the 499–651 nm wavelength range. mCherry was excited at 561 nm, and the emission spectrum was recorded in channel mode at 595–615 nm. The protoplasts imaged in lambda mode were subjected to linear unmixing using Zen software (https://www.zeiss.com). Reference spectra of GFP and chlorophyll were captured from Arabidopsis roots expressing the GFP protein and from leaf tissues of plants not expressing GFP, respectively. The composite spectra from protoplasts expressing GFP fusion proteins were unmixed using the reference spectra of GFP and chlorophyll to obtain the images shown in the figures. To assess co-localization, Pearson correlation coefficients were calculated from images of 16 protoplasts using the Coloc2 plugin of ImageJ software v1.51 (NIH; https://imagej.nih.gov).

## Results

### Localization of SCY1 and SCY2

**Fig. 1.**
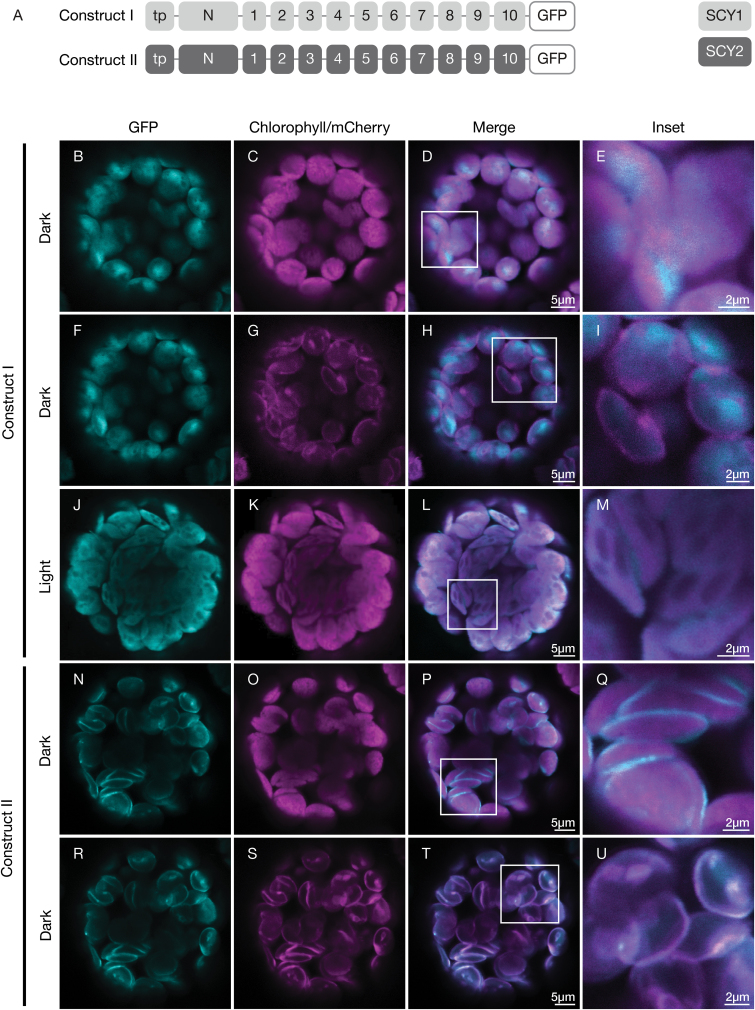
Localization of SCY1 and SCY2 in transfected protoplasts. (A) Structural representation of GFP (green fluorescent protein) constructs I and II. Individual transmembrane domains (1–10), the N-terminal regions (N) of the mature proteins, and the transit peptides (tp) are indicated. (B–U) Protoplasts from 3-week-old wild-type (Ws ecotype) plants were transfected with construct I (B–M) or construct II (N–U) together with the inner-envelope marker TIC20-mCherry, and incubated under either dark (B–I, N–U) or light (J–M) conditions for 12 h before imaging. The images show GFP fluorescence (cyan), chlorophyll fluorescence (magenta in C, K, O), TIC20-mCherry fluorescence (magenta in G, S), merged images, and higher magnification views (inset) of the boxed areas.

To localize SCY1 and SCY2 in chloroplasts, we imaged fluorescent fusion proteins consisting of full-length SCY1 (construct I) and SCY2 (construct II) proteins with C-terminal GFP fusions (Fig 1A) in transfected Arabidopsis leaf protoplasts. Constructs encoding these fusion proteins were individually co-transfected along with a construct encoding a TIC20-mCherry fusion protein, a marker for the inner-envelope membrane. Following transfection, the protoplasts were incubated under dark conditions for 12 h before GFP fluorescence signals were analysed by confocal microscopy. It is well established that SCY1 is confined to the thylakoids in mature chloroplasts. However, for SCY1-GFP, the fluorescent signal did not overlap with chlorophyll autofluorescence (Pearson correlation coefficient *r*=0.021 ± 0.26), which marks the location of the thylakoids (Fig. 1B–E), nor did it coincide with the TIC20-mCherry signal, located in the envelope (Fig. 1F–I). Instead, the fusion protein appeared to accumulate in the stroma of the chloroplasts, with a distribution that resembled that of a small subunit of Rubisco (SSU)–GFP fusion protein (Supplementary Fig. S2). We hypothesized that the dark-incubated chloroplasts might lack a light-dependent factor or condition necessary for SCY1 integration in the thylakoids, such as a strong proton motive force. To test this, we incubated the protoplasts in light for 12 h following transfection. In light-exposed protoplasts, the SCY1-GFP signal overlapped almost completely with the chlorophyll autofluorescence (*r*=0.74 ± 0.079; Fig. 1J–M). The same result was achieved by exposing transfected protoplasts incubated in the dark for 12 h to light for 5 h before imaging (data not shown). In contrast, in protoplasts transfected with SSU-GFP, the distribution of GFP fluorescence did not overlap with that of chlorophyll autofluorescence following incubation under light conditions (Supplementary Fig. S2).

SCY2 was previously reported to be preferentially associated with the inner-envelope membrane, although some association with thylakoids could not be ruled out (Skalitzky *et al.*, 2011). For SCY2-GFP, we found little or no overlap between the GFP fluorescence and chlorophyll autofluorescence (*r*=0.26 ± 0.07; Fig. 1N–Q). Instead, the fluorescent signal appeared to be largely confined to the periphery of the chloroplasts and coincident with the TIC20-mCherry signal (*r*=0.84 ± 0.04; Fig. 1R–U). Therefore, it appears that SCY2 is associated almost exclusively with envelope in mature chloroplasts. Although this technique does not allow us to resolve inner- and outer-envelope localizations, SCY2 is almost certainly associated with the inner envelope based on other studies (Skalitzky *et al.*, 2011; Li *et al.*, 2015, 2017).

### Identification of regions required for differential targeting to the envelope or thylakoids

**Fig. 2.**
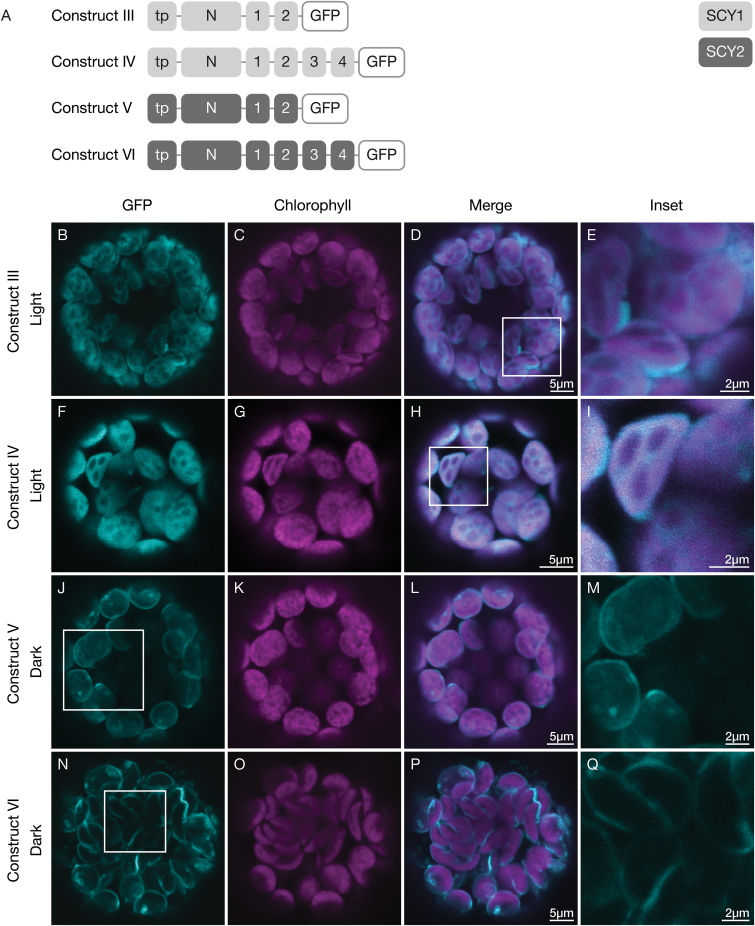
Localization of truncated SCY1 and SCY2 proteins in transfected protoplasts. (A) Structural representation of GFP (green fluorescent protein) constructs III, IV, V, and VI. Numbers indicate individual transmembrane domains; N, N-terminal region; tp, transit peptide. (B–Q) Protoplasts from 3-week-old wild-type (Ws ecotype) plants were transfected with construct III (B–E), IV (F–I), V (J–M), or VI (N–Q), and incubated under either light (B–I) or dark (J–Q) conditions for 12 h before imaging. The images show GFP fluorescence (cyan), chlorophyll fluorescence (magenta), merged images, and higher magnification views (inset) of the boxed areas.

To identify regions responsible for the differential targeting of SCY1 and SCY2, we used C-terminal truncations to determine the minimum portion of each protein required to replicate the behavior of the full-length protein. Constructs encoding truncated versions of either SCY1 or SCY2 fused to GFP (Fig. 2A) were expressed in protoplasts. We found that expressing truncated SCY1-GFP proteins that contained the N-terminal region plus either the first two TMDs (construct III) or first four TMDs (construct IV), resulted in accumulation in the stroma under dark conditions (Supplementary Fig. S3). Increased association with thylakoid membranes, as indicated by overlap with chlorophyll auto-fluorescence, was seen in light-exposed protoplasts for both construct III (Fig. 2B–E) and construct IV (Fig. 2F–I). The extent of overlap was approximately the same for both constructs; therefore, TMD3–4 may not be needed for light-dependent thylakoid localization. The truncated version of SCY2-GFP protein containing the N-terminal region and first two TMDs (construct V) was associated with both the periphery and internal regions of the chloroplasts (Fig. 2J–M), as indicated by the more diffuse signal. The version truncated after the first four TMDs (construct VI) was preferentially associated with the periphery (Fig. 2N–Q). These results indicate that the region containing TMD3–4 of SCY2 is required for high-fidelity envelope targeting.

**Fig. 3.**
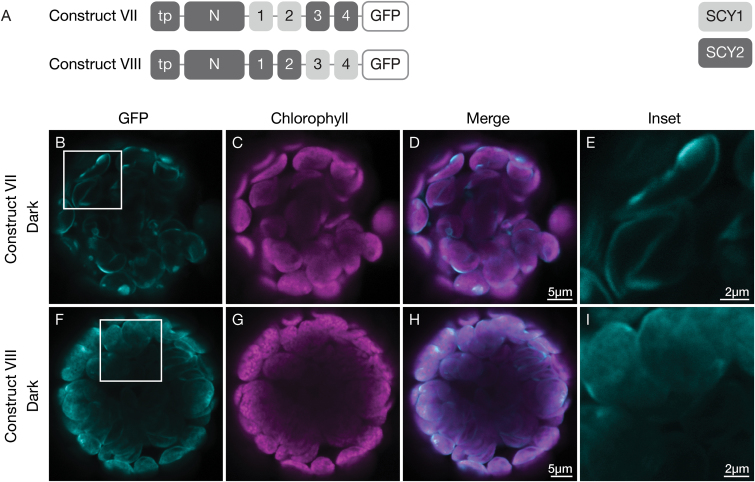
Localization of chimeric truncated SCY1-SCY2 proteins in transfected protoplasts. (A) Structural representation of GFP (green fluorescent protein) constructs VII and VII. Numbers indicate individual transmembrane domains; N, N-terminal region; tp, transit peptide. (B–I) Protoplasts from 3-week-old wild-type (Ws ecotype) plants were transfected with construct VII (B–E) or construct VIII (F–I), and incubated under dark conditions for 12 h before imaging. The images show GFP fluorescence (cyan), chlorophyll fluorescence (magenta), merged images, and higher magnification views (inset) of the boxed areas.

To further investigate the role of SCY2 TMDs in envelope targeting, we generated constructs where TMD1–2 or TMD3–4 of SCY2 were replaced by the corresponding TMDs from SCY1 (Fig. 3A). Replacing TMD1–2 of SCY2 (construct VII) did not disrupt envelope targeting (Fig 3B–E); however, replacing TMD3–4 of SCY2 (construct VIII) resulted in a more diffuse signal. GFP fluorescence associated with the periphery decreased and GFP fluorescence associated with internal regions of the chloroplast increased (Fig. 3F–I). The latter result is similar to that seen when TMD3–4 were deleted from SCY2 (construct V). The results of these experiments indicate that the sequence composition of SCY2 TMD3–4 and the intervening loop, and not just the presence of additional hydrophobic domains, is likely to be important for targeting truncated SCY2 proteins to the inner envelopes of the chloroplasts.

### The N-terminal region of SCY1 contains elements necessary for sorting to the stroma

SCY1 and SCY2 targeting is likely to involve different mechanisms. Based on the distribution of SCY1-GFP under different light conditions, targeting of imported SCY1 involves two distinct, sequential steps. The first step, which is light-independent, involves passage of the protein from the TIC complex into the stroma. Given the highly hydrophobic nature of SCY1, this step probably involves interactions with other proteins to form a stable stromal intermediate. The second step involves integration into the thylakoid membranes and is enhanced by light. Distinct steps are not as apparent in the targeting of SCY2, and there is no evidence of the formation of a persistent stromal intermediate. Therefore, to identify determinants that contribute to differential sorting of these proteins, we focused on identifying sequence elements that contribute to accumulation of a stroma-localized fusion protein under dark conditions.

**Fig. 4.**
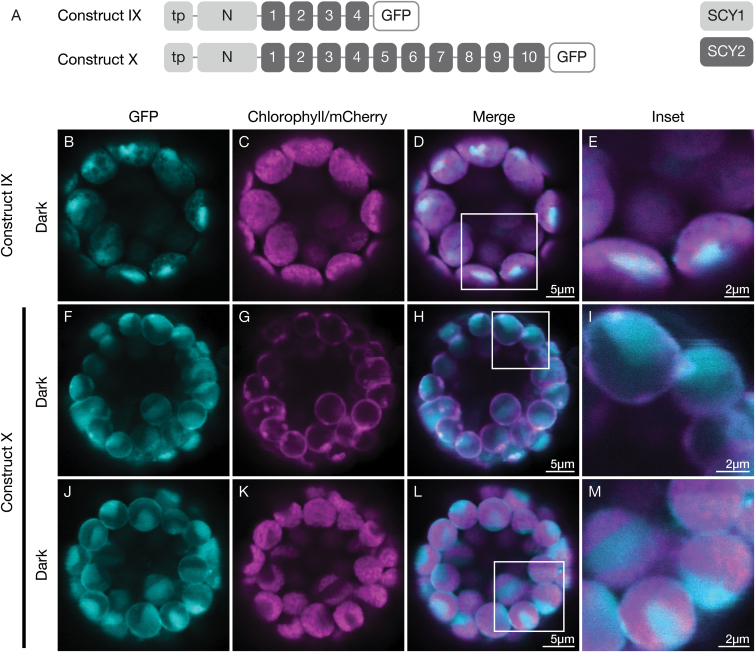
Effect of the SCY1 N-terminal region and transit peptide on SCY2 targeting in transfected protoplasts. (A) Structural representation of GFP (green fluorescent protein) constructs IX and X. Numbers indicate individual transmembrane domains; N, N-terminal region; tp, transit peptide. (B–M) Protoplasts from 3-week-old wildtype (Ws ecotype) plants were transfected with construct IX (B–E) or construct X (F–M) together with the inner-envelope marker TIC20-mCherry, and incubated under dark conditions for 12 h before imaging. The images show GFP fluorescence (cyan), chlorophyll fluorescence (magenta in C, K), TIC20-mCherry fluorescence (magenta in G), merged images and higher magnification views (inset) of the boxed areas.

We first considered the role of the N-terminal region, which includes the transit peptide that is cleaved following import and the region of the mature protein N-terminal to the first TMD (designated here as the N region). We created constructs encoding chimeric SCY2-GFP proteins where the N-terminal region of SCY2 was replaced with the N-terminal region of SCY1 (Fig. 4A). Although SCY2 truncated after the first four TMDs (construct VI) was reliably targeted to the envelope, the corresponding chimeric protein (construct IX) accumulated in the stroma (Fig 4B–E). This suggests that determinants in the SCY1 N-terminal region that direct proteins to the stroma are dominant over any targeting signals that may be associated with SCY2 TMDs. Alternatively, addition of the SCY1 N-terminal region may have resulted in a protein that was incapable of integrating into the envelope. To further test the effect of the SCY1 N-terminal region, we fused it to all 10 TMDs of SCY2 (construct X). The resulting fusion protein partitioned between the stroma and the inner envelope, as indicated by overlap with TIC20-mCherry signals (Fig 4F–I), but, as with construct IX, a significant portion ended up in the stroma (Fig. 4J–M). Therefore, addition of the SCY1 N-terminal region is not completely incompatible with membrane integration; and instead, it appears that determinants in the SCY1 N-terminal region are capable of redirecting individual proteins that are normally targeted to the envelope. The fact that the fusion proteins partitioned between two locations provided us with a sensitive assay, since we could easily detect shifts either towards or away from either location. Therefore, we based subsequent experiments on constructs encoding all 10 TMDs of SCY2.

### Multiple sub-regions of the SCY1 N-terminal region have elements that enhance accumulation in the stroma

**Fig. 5.**
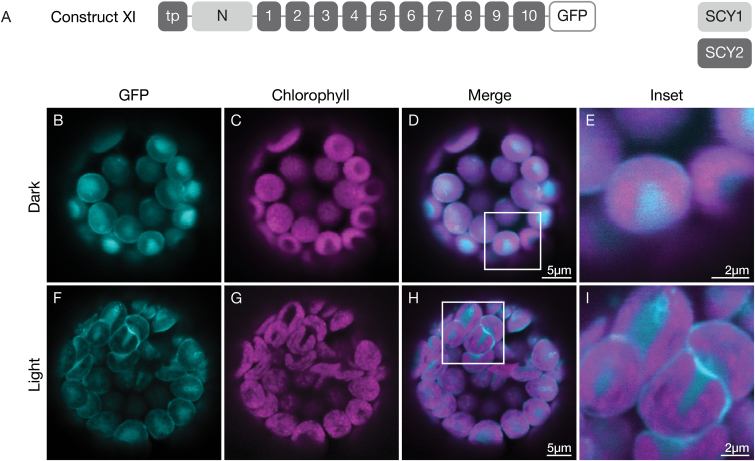
Effect of the SCY2 transit peptide on displacement of SCY2 TMDs into the stroma in transfected protoplasts, mediated by the N-terminal region of mature SCY1. (A) Structural representation of GFP (green fluorescent protein) construct XI. Numbers indicate individual transmembrane domains; N, N-terminal region; tp, transit peptide. (B–I) Protoplasts from 3-week-old wildtype (Ws ecotype) plants were transfected with construct XI, and incubated under dark (B–E) or light (F–I) conditions for 12 h before imaging. The images show GFP fluorescence (cyan), chlorophyll fluorescence (magenta), merged images, and higher magnification views (inset) of the boxed areas.

To identify the relevant determinants in the N-terminal region, we first considered the effect of the transit peptide. A construct (construct XI) was generated that was identical to construct X but included a sequence encoding the SCY2 transit peptide in place of the SCY1 transit peptide (Fig. 5A). As with construct X, the fusion protein partitioned between the envelope and stroma under dark conditions (Fig. 5B–E). Therefore, we conclude that stromal targeting is a function of the N region (N-terminal region of the mature protein) and the transit peptide does not contribute. To test whether this region also confers an ability to route SCY2 TMDs to the thylakoids, we also incubated protoplasts transfected with construct XI under light conditions, but failed to detect significant reduction of the stromal pool or association of fusion protein with the thylakoids (Fig. 5F–I).

**Fig. 6.**
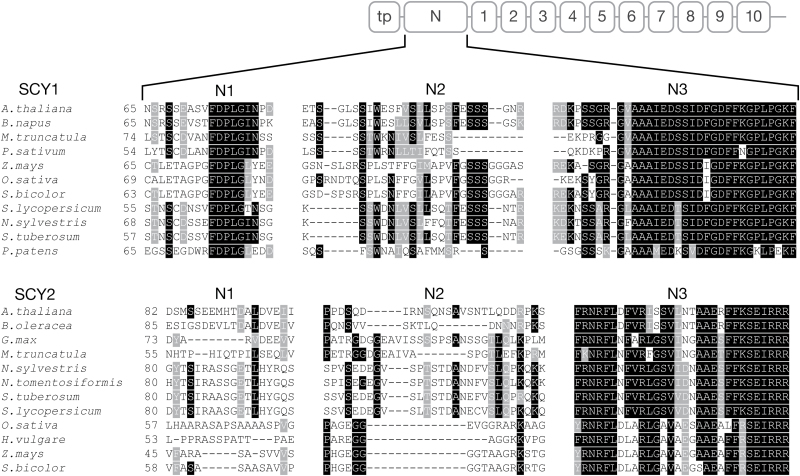
Alignment of amino acid sequences of N-terminal regions of mature SCY1 and SCY2 from multiple plant species. Three sub-regions (N1, N2, N3) were delineated based on the degree of amino acid conservation. Amino acids highlighted in black are conserved. Similar amino acids are shown in gray.

To identify regions that may contain determinants within the N regions, we aligned the amino acid sequences of the N regions of SCY1 (amino acids 65–143) and SCY2 (amino acids 82–156) from multiple plant species (Fig. 6). Based on this alignment, we divided each N region into three sub-regions. Sub-region 1 (N1), which consists of 18 amino acids in the Arabidopsis proteins, is not highly conserved in SCY2, but contains a small (five-amino acid) motif of highly conserved residues in SCY1. Sub-region 2 (N2), which consists of 27 (SCY1) or 26 (SCY2) amino acids in Arabidopsis, has some conserved residues but a lower degree of sequence conservation overall. Sub-region 3 (N3) consists of 33 (SCY1) or 30 (SCY2) amino acids in Arabidopsis. The SCY1 and SCY2 N3 sequences are not similar, but they are highly conserved between species.

**Fig. 7.**
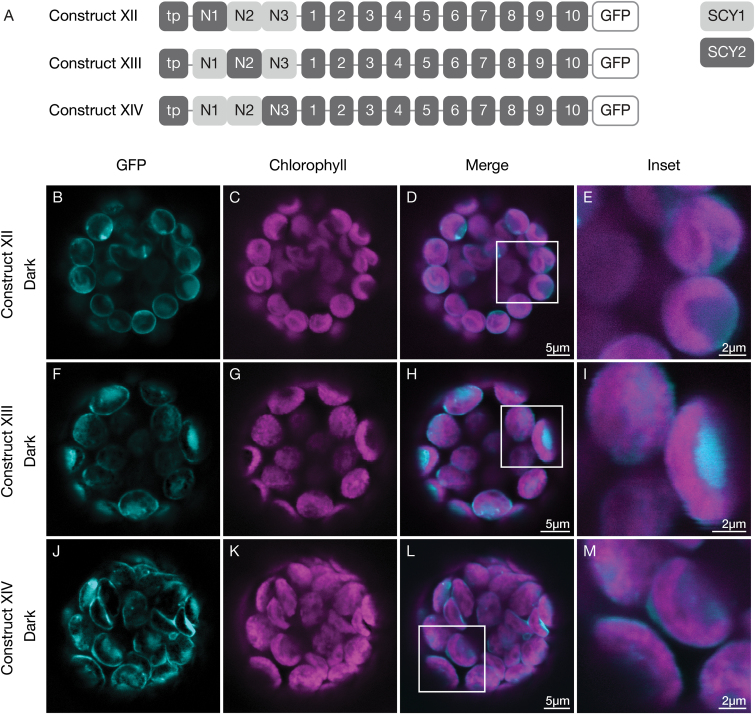
Contributions of different sub-regions to the effect of the N-terminal region of SCY1 on SCY2 localization in transfected protoplasts. (A) Structural representation of GFP (green fluorescent protein) constructs XII, XIII, and XIV. Numbers indicate individual transmembrane domains; N, N-terminal region; tp, transit peptide. (B–M) Protoplasts from 3-week-old wildtype (Ws ecotype) plants were transfected with construct XII (B–E), construct XIII (F–I), or construct XIV (J–M), and incubated under dark conditions for 12 h before imaging. The images show GFP fluorescence (cyan), chlorophyll fluorescence (magenta), merged images, and higher magnification views (inset) of the boxed areas.

To assess the contributions of each sub-region of SCY1, we used construct XI as a base and generated constructs XII, XIII, and XIV, which systemically eliminated the SCY1 N1, N2, or N3 sub-regions by substituting the corresponding SCY2 sequence (Fig. 7A). Substitution of sub-region N2 (construct XIII), the least conserved sub-region, resulted in little or no loss of stromal accumulation (Fig. 7F–I). Substitution of either sub-region N1 (construct XII) or sub-region N3 (construct XIV), however, resulted in an observable decrease in stromal accumulation and increase in envelope localization (Fig. 7B–E, J–M). We conclude that important determinants for stromal accumulation are likely to be associated with sub-regions N1 and N3 of SCY1, which is consistent with the high degree of sequence conservation in these regions.

### Sub-region N1 of SCY1 may mediate an interaction with SRP pathway components

**Fig. 8.**
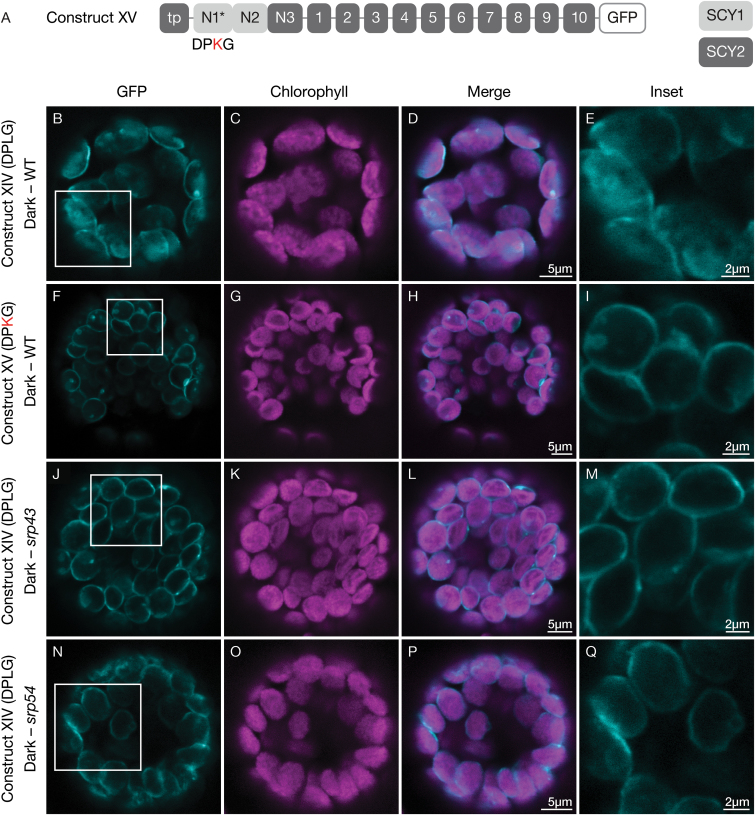
Contributions of the L18-like motif and SRP pathway components to the effect of the N-terminal domain of SCY1 on SCY2 localization in transfected protoplasts. (A) Structural representation of GFP (green fluorescent protein) construct XV. Numbers indicate individual transmembrane domains; N, N-terminal region; tp, transit peptide. The amino acid altered in the mutant protein is indicated in red. (B–Q) Protoplasts from 3-week-old wild-type (B–I), *srp43* mutant (J–M), or *srp54* mutant (N–Q) plants were transfected with construct XIV (B–E, J–Q) or construct XV (F–I), and incubated under dark conditions for 12 h before imaging. The images show GFP fluorescence (cyan), chlorophyll fluorescence (magenta), merged images, and higher magnification views (inset) of the boxed areas.

For further investigations, we focused on sub-region N1 of SCY1. In this sub-region, the small, highly conserved amino acid motif DPLG (Fig 6A) was an obvious candidate as a targeting determinant. The same motif is present in LHCPs in the region between the second and third TMDs and forms the core of what is known as the L18 sequence (DeLille *et al.*, 2000). This region is necessary for binding by the stromal chaperone and SRP pathway component SRP43 (Tu *et al.*, 2000). Interactions with SRP43 are necessary to prevent aggregation of the highly hydrophobic LHCPs in the stroma (Falk and Sinning, 2010). Previous studies have shown that the amino acid motif DPLG is highly conserved in LHCPs and mutation of leucine (L) to lysine (K) abolishes SRP43 binding and impairs integration into the thylakoid membrane (Stengel *et al.*, 2008). To test whether this motif might act similarly in SCY1, we changed the sequence encoding DPLG to one encoding DPKG in the context of construct XIV, where the N1 sub-region should be the primary source of determinants for stromal accumulation; this produced construct XV (Fig. 8A). The resulting fusion protein was almost exclusively associated with envelopes (compare the strong envelope signals in Fig. 8F–I with the more diffuse signals in 8B–E). To confirm that SRP43 is involved in stromal accumulation, we expressed construct XIV, with an intact DPLG motif, in protoplasts isolated from the leaves of *srp43* mutants. In the absence of SRP43, the fusion protein localized primarily to the envelope (Fig. 8J–M). Finally, to test whether other components of the SRP pathway, such as SRP54, which interacts with SRP43, are involved, we expressed construct XIV in protoplasts isolated from the leaves of *srp54* mutants. In the absence of SRP54, we found that the fusion proteins were also largely associated with the envelope (Fig. 8N–Q). As a control for non-specific effects in mutant protoplasts, we localized GFP fused to PLSP1, a thylakoid protein that is known to be targeted via an SRP-independent process (Endow *et al.*, 2015), in wild-type, *srp43*, and *srp54* protoplasts. PLSP1 has one TMD, a large C-terminus exposed to the thylakoid lumen, and a smaller N-terminus exposed to the stroma. To fuse GFP to the stroma-exposed N-terminus of the mature protein, a sequence encoding GFP was inserted downstream of the sequence encoding the transit peptide (Supplementary Fig. S4A). When this construct was expressed in wild-type and mutant cells, we found that the GFP fluorescence overlapped with the chlorophyll fluorescence in the wild-type (Fig. S4B–E), *srp43* (Fig. S4F–I), and *srp54* (Fig. S4J–M) cells, indicating that thylakoid integration *per se* is not disrupted in *srp* mutant cells. These results suggest that the SRP pathway is likely to be involved in SCY1 targeting, with the conserved amino acids in the N1 region playing a role in recruitment of SRP43.

## Discussion

### SCY1 and SCY2 are targeted by different mechanisms

Localization of full-length SCY1 and SCY2 proteins indicated that they require different physiological conditions for membrane association. Specifically, light is required for association of SCY1 with the thylakoid membranes, but not for association of SCY2 with the inner envelope. A truncated SCY1 protein that only contains the N region, TMD1–2, and the intervening loop also requires exposure to light for membrane association (Fig. 2B–E), suggesting that these regions of the protein contribute to the light requirement. Both SCY1 and SCY2 proteins contain a 39-amino acid hydrophilic loop between TMD1 and TMD2, which forms the plug that occupies the central channel of the translocase (Supplementary Fig. S1) (Park *et al.*, 2014). In the case of SCY1, that is the longest loop that must be translocated across the thylakoid membrane to achieve the correct orientation. Given the size of the loop and presence of charged residues, the translocation process is likely to require energy, which may be provided in the form of a proton motive force and/or ATP. Li *et al.* (2017) recently demonstrated that ionophores, which dissipate the proton motive force, impair localization of SCY1. This is consistent with our observation that light is required, because the proton motive force is stronger in the light than in the dark (Takizawa *et al.*, 2007). We found that light exposure for several hours following the dark incubation period was also effective in allowing SCY1 to become associated with thylakoids. Thylakoid association occurred even if the protein synthesis inhibitor cycloheximide was added at the time of light exposure (data not shown), suggesting that a pool of productive intermediates forms in the stroma in the dark.

For SCY2, the predicted orientation, with N- and C-termini exposed to the stroma, could be achieved by either of two mechanisms. This orientation was confirmed by previous yeast 2-hybrid studies on the membrane complex formed by SCY2 and SECE2 (Li *et al.*, 2015) and would put the loop constituting the plug domain (between TMD1 and 2) in the intermembrane space. If TMD1 and TMD2 diffuse laterally from the TIC into the bilayer of the inner envelope via the stop-transfer pathway, no translocation of the intervening loop would be needed. However, this model is difficult to reconcile with the observation that truncated SCY2 proteins consisting of the N region and the first two TMDs readily enter the stroma. Alternatively, if TMD1, TMD2, and the intervening loop transit through the TIC complex, they would need to be inserted via a post-import pathway. This would require an envelope-based translocase capable of accepting substrates from the stroma, such as the SEC2 system, which was recently shown to mediate integration of the inner-envelope protein TIC40 from the stromal side (Li *et al.*, 2017). In this case, energy may be supplied by ATP hydrolysis by the SECA2 component, and the process would be azide sensitive. Further tests will be needed to definitively distinguish between the two possible mechanisms of insertion for TMD1 and TMD2.

### Characteristics of TMDs and their relationship to targeting

**Fig. 9.**
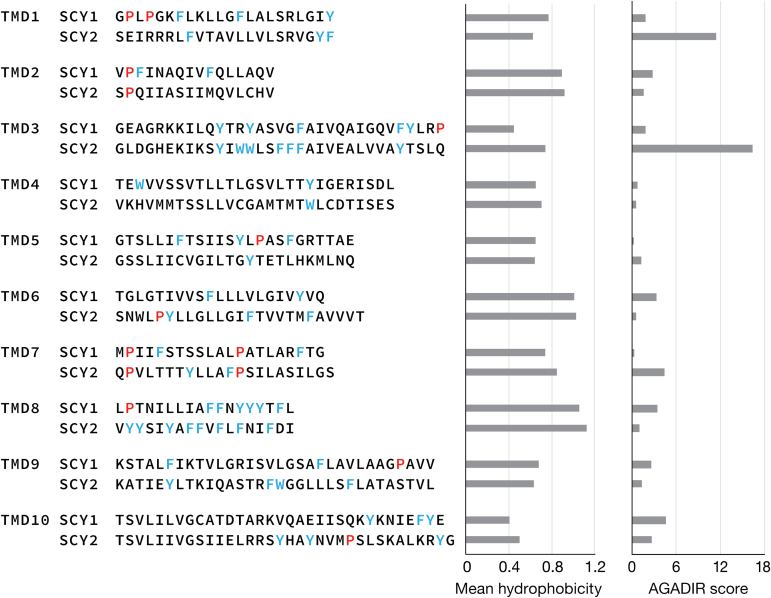
Comparisons of the amino acid sequences and properties of individual transmembrane domains (TMDs) of SCY1 and SCY2. The predicted TMDs were identified by aligning SCY1, SCY2, and *E. coli* SecY sequences and identifying the regions that correspond to the *E. coli* TMDs. Aromatic hydrophobic amino acids are indicated in blue and prolines in red. The mean hydrophobicity of residues in each TMD was calculated using the HELIQUEST web server (http://heliquest.ipmc.cnrs.fr). The Adagir score, which predicts the helical behavior of peptides in aqueous solutions, was obtained for each TMD using the Agadir calculator (http://agadir.crg.es).

TMDs other than TMD1 and TMD2 may play a role in targeting of SCY2 because truncated SCY2 proteins that include TMD1–4 are targeted more efficiently to the envelope (Fig. 2N–Q) than truncated proteins that include only TMD1–2 (Fig. 2J–M). The effect is primarily a function of sequences in SCY2’s TMD3, TMD4, and the intervening loop, and not the number of TMDs, because the comparable SCY1 domains cannot substitute for the SCY2 domains (Fig. 3F–I). We compared all of the TMDs of SCY1 and SCY2 with regard to their amino acid content, mean hydrophobicity, and helical propensity in aqueous solutions. The predicted TMDs (obtained by alignment with the *E. coli* SecY sequence, as described in the Materials and Methods) varied in length from 16 (TMD2) to 33 (TMD3, TMD10) amino acids (Fig. 9). Froehlich and Keegstra (2011) have previously observed that proline residues are often absent in the TMD of inner-envelope proteins, and stop-transfer TMDs often have a relatively high content of clustered tryptophan and phenylalanine residues. There does not appear to be a significant difference in the number or position of proline residues in SCY1 and SCY2 TMDs. However, TMD3 and TMD8 of SCY2 each contain five tryptophan or phenylalanine residues in the center of the TMD. Although strong hydrophobicity has not been noted as an important feature of inner-envelope proteins in general (Froehlich and Keegstra, 2011), it is one of the important features of TMDs that integrate via a stop-transfer mechanism in mitochondria (Meier *et al.*, 2005). For most of the TMDs, the mean hydrophobicities are fairly similar in SCY1 and SCY2; however, TMD3 is significantly more hydrophobic in SCY2 than in SCY1 (Fig. 9). Agadir scores, which predict the helical behavior of monomeric peptides in aqueous solution, were also calculated for each TMD and were largely equivalent with the exception of TMD1 and TMD3 of SCY2 (Fig. 9). The elevated Agadir scores of these two TMDs suggest that TMD1 and TMD3 of SCY2 might assume a more compact configuration than the corresponding TMDs of SCY1 when they are transiting aqueous channels. We conclude that TMD3 of SCY2 has a unique set of characteristics that distinguish it from TMD3 of SCY1. The significance of these biochemical differences may be more apparent once we have a better understanding of the nature of the TIC import channel and mechanism for TMD insertion. We propose that, within SCY2, TMD3 is the single best candidate for integration by a stop-transfer mechanism.

### Role of the N-terminal regions in SCY targeting

For SCY1, our results indicate that the N-terminal region plays a role in recruiting SRP components. A seven-amino acid segment containing the SRP43 interaction motif DPLG is present and highly conserved in a variety of plant species (Fig. 6), and addition of a SCY1 segment (N1 and N2) that includes the DPLG motif is sufficient to displace SCY2 proteins that normally localize to the envelope into the stroma (Fig. 7F–M). The importance of the DPLG motif for this effect was shown through mutagenesis to DPKG, which had previously been shown to abolish interactions with SRP43 (Stengel *et al.*, 2008) and which abolished stroma localization of SCY2 proteins in our experiments (Fig. 8F–I). We also showed that displacement into the stroma was SRP43- and SRP54-dependent, which directly implicates the SRP pathway in this process. The recently published observation that integration of SCY1 into the thylakoids is partially blocked by antibodies against the ALB3 translocase is also consistent with delivery of SCY1 via the SRP pathway (Li *et al.*, 2017). Based on these results, we suggest membrane proteins other than the LHCPs, such as SCY1, may depend on this pathway for efficient targeting to the thylakoid membrane.

Other regions of the N-terminal region of SCY1 also contribute to targeting. Displacement of the SCY2 test protein with 10 TMDs into the stroma still occurred when the N1 sub-region of SCY1 was absent and the N2 and N3 sub-regions were present, although it was strongest when both of the conserved sub-regions (N1 and N3) were present (Fig. 7F–I). The high degree of sequence conservation associated with the N3 region suggests that this may be an interaction site of some kind. Further experiments will be needed to determine whether SRP components interact with this region or if other protein mediators are likely to be involved.

For SCY2, the N1 and N2 sub-regions of the N-terminal region are not conserved, and avoiding interactions with SRP components may be the most critical feature of these sub-regions of SCY2. On the other hand, the N3 region of SCY2, while different in sequence from SCY1, shows a high degree of sequence conservation in different plant species. We did not specifically test the function of this region but hypothesize that it may play a role in interactions that support efficient envelope integration.

### Summary model

**Fig. 10.**
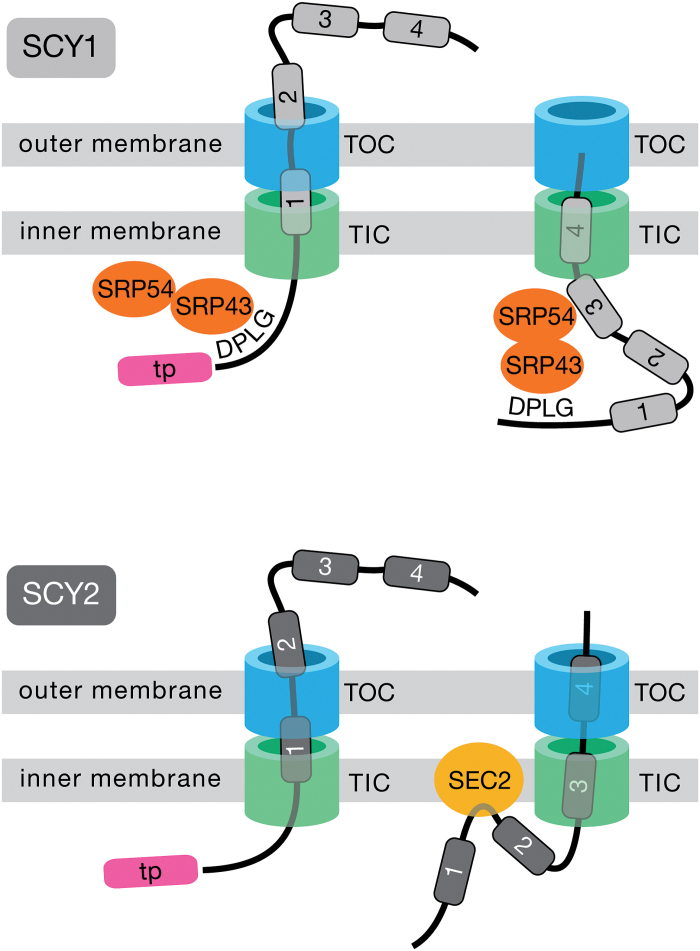
A model comparing the initial steps in targeting of SCY1 and SCY2. For simplicity, truncated proteins with four transmembrane domains (TMDs, numbered segments) rather than 10 are shown. For SCY1, the DPLG motif is exposed when the protein exits the TIC complex and is recognized by components of the SRP pathway (SRP43, SRP54). SRP components would also bind to the TMDs as they exit the TIC complex. For SCY2, which lacks the DPLG motif, SRP components are not recruited. Particular TMDs, such as TMD3, may transit the TIC complex in a manner that allows them to diffuse laterally into the bilayer, thereby anchoring the protein in the membrane and allowing other TMDs, such as TMD1 and TMD2, to be inserted by the SEC2 system. tp, transit peptide. (This figure is available in color at *JXB* online).

We have generated the following hypothetical model to explain our results (Fig. 10). We propose that membrane-specific sorting reflects the outcome of a kinetic competition between two processes. The first process (import) involves exit of TMDs from the TIC channel via translocation, and the second (membrane insertion) involves release of TMDs from the TIC channel via lateral diffusion. Events that occur soon after the N-terminus of the mature protein exits the TIC channel and is exposed in the stroma may impact the relative rate of these processes. In the case of SCY1, the most N-terminal region of the mature protein contains recognition motifs that recruit SRP components to the vicinity of the TIC exit (Fig. 10A). Binding of these targeting factors to emerging TMDs might kinetically enhance the import process, ensuring that membrane insertion does not occur. In addition, interactions with SRP components might physically block interactions with factors needed for post-import sorting to the envelope, thereby ensuring that SCY1 is routed to the thylakoids.

In the case of SCY2, which lacks the DPLG motif, SRP components would not be recruited to the exposed N-terminus and TIC exit. SCY2 TMDs may transit the TIC with slower kinetics, allowing time for TMD3 (or other TMDs) to be released into the inner-envelope bilayer by lateral diffusion. Once this has occurred for any single TMD, the protein will be anchored in the membrane, and other regions may have increased opportunities to interact with membrane-associated factors, some of which may support post-import insertion into the envelope, such as the SEC2 complex (Fig. 10B). Thus, we imagine that the situation in chloroplasts is similar to that in mitochondria, where different TMDs can insert via different mechanisms. For example, for Mdl1, which consists of six TMDs, TMD1 and TMD2 are released laterally within the lipid bilayer by the Tim23 translocase complex, but TMD3 and TMD4 are first imported into the matrix by Hsp70 and exported into the lipid bilayer by Oxa1 translocase (Bohnert *et al.*, 2010).

In conclusion, we have identified some of the key elements within the SCY proteins that may play a role in directing them to either the inner envelope or thylakoid membrane. The N-terminal region of SCY1 contains a targeting determinant that allows interaction with components of the SRP pathway. For envelope localization of SCY2, particular TMDs appear to play an important role. However, efficient integration of SCY2 in the envelope occurs only in the absence of competing signals in other regions. Our findings may be of particular interest to investigators seeking to engineer other capabilities into the membranes of chloroplasts. Two recent studies have focused on strategies to target cyanobacterial bicarbonate transporters such as BicA and SbtA to the inner envelope to enhance the photosynthesis in C_3_ plants (Rolland *et al.*, 2016; Uehara *et al.*, 2016). In Uehara *et al.* (2016), localization in the inner envelope was achieved by fusing the transporters to the inner-envelope protein COR413IM1. The COR413IM1 TMDs probably act as an anchor in this case, enhancing the ability of envelope-based integrators to engage the bicarbonate transporter TMDs. In the study by Rolland *et al.* (2016), chloroplast localization and membrane integration were achieved by fusing the extended N region of the inner-envelope proteins PLGG1 and HP59 to the cyanobacterial transporters. This study shows that the N region is sufficient for targeting these proteins to chloroplasts; however, part of the chimeric protein pool was found to localize inside chloroplasts, rather than just in the chloroplast envelope. To explain this, Rolland *et al.* (2016) suggested that once a chimeric protein has reached the chloroplast, the sequence of the cyanobacterial transporter determines which internal membrane the protein localizes to, hypothesizing that this sub-compartment targeting could be mediated by the protein’s TMDs. These findings are in line with our observation that chimeric proteins with the N region of SCY2 and SCY1 TMD1–4 were successfully imported into chloroplasts and targeted to the thylakoids (data not shown). We conclude that in order to achieve high-fidelity targeting to particular membranes in chloroplasts, the dynamics of the integration process and contributions of both the TMDs and N regions must be taken into account.

## Supplementary data

Supplementary data are available at *JXB* online.

Table S1. List of oligonucleotides used for PCR genotyping and GFP construct generation.

Fig. S1. Molecular modeling of SecY and SCY proteins.

Fig. S2. Targeting of small subunit of Rubisco in transfected protoplasts under light and dark conditions.

Fig. S3. Localization of truncated SCY1 proteins in transfected protoplasts under dark conditions.

Fig. S4. Targeting of PLSP1 to the thylakoids in wild-type and *srp* mutants.

## Supplementary Material

Supplementary_Table_S1_Supplementary_Figures_S1_S4

## Abbreviations:

ALB3: ALBINO 3
HSP: heat-shock protein
LHCP: light-harvesting chlorophyll *a,b*-binding protein
PLSP1: plastidic type 1 signal peptidase 1
SRP: signal-recognition particle
SSU: small subunit of Rubisco
TMD: transmembrane domain
TOC: translocon at the outer envelope membrane of chloroplasts
TIC: translocon at the inner envelope membrane of chloroplasts.
