# Supplementary material for: Sorting of SEC translocase SCY components to different membranes in chloroplasts
Source: J Exp Bot. 2017 Sep 13;68(18):5029–43. doi: 10.1093/jxb/erx318 (PMC5853536; doi:10.1093/jxb/erx318)
Supplement: Supplementary_Table_S1_Supplementary_Figures_S1_S4 [file erx318_suppl_supplementary_table_s1_supplementary_figures_s1_s4.pdf]

Table S1. Oligonucleotides used for PCR genotyping and GFP construct generation

Oligonucleotides used for PCR genotyping

Allele	Forward	Reverse
srp43	TGCAGTTCGGGAGGAGAATT	TAGCATCTGAATTTCATAACCAATCTCGATACAC
SRP43	TGCAGTTCGGGAGGAGAATT	ATAGTGTCCCCAAAAGGCCA
srp54	ATATTGTTGGCTGGGCTCCA	AACGTCCGCAATGTGTTATTAAGTTGTC
SRP54	ATATTGTTGGCTGGGCTCCA	TGAAGCCTCCCTGCAGTATC

Oligonucleotides used for GFP construct generation

Construct	Forward	Reverse
I	TTGGTACCATGATAACGGTAAGCGAAGTTTC	TTACTAGTTGGATCATACTTGTCAAGCTCG
II	GATCTAGACTCGAGGGTACCATGAATTCCTCTCAAG	ACTTTTGCTCACTAGTTACGCCATATCTCTTT
III	GATCTAGACTCGAGGGTACCATGATAACGGTAAGC	ACTTTTGCTCACTAGTACCTTCTTTTTTCTG
IV	GATCTAGACTCGAGGGTACCATGATAACGGTAAGC	ACTTTTGCTCACTAGTCAGATCAGAGATTCT
V	TTGGTACCGTCGACCCCGGGCTCCTCATCCTCTCGATGAAT	TTACTAGTGCATGCGGCCGCTTCCTTTCGCAGTTTCACC
VI	TTGGTACCGTCGACCCCGGGCTCCTCATCCTCTCGATGAAT	TTACTAGTGCCATGACCAAATCCAGATTC
VII a	GATCTAGACTCGAGGGTACCATGAATTCCTCTCAAG	CCCAAGAAGCTTCAGCCTCCTCCTTATCTC
VII b	GAGATAAGGAGGAGGCTGAAGCTTCTTGGG	GTGACCATCTAAGCCACCTTCTTTTTTCTG
VII c	CAGAAAAAAGAAGGTGGCTTAGATGGTCAC	TTACTAGTGCCATGACCAAATCCAGATTC
VIII a	GATCTAGACTCGAGGGTACCATGAATTCCTCTCAAG	TTTTCTACCAGCTTCTTCCTTTCGCAGTTT
VIII b	AAACTGCGAAAGGAAGAAGCTGGTAGAAAA	ACTTTTGCTCACTAGTCAGATCAGAGATTCT
IX a	GATCTAGACTCGAGGGTACCATGATAACGGTAAGC	AGCCGTTACAAACAAAAACTTCCCTGGCAA
IX b	TTGCCAGGGAAGTTTTTGTTTGTAACGGCT	TTACTAGTGCCATGACCAAATCCAGATTC
X a	GATCTAGACTCGAGGGTACCATGATAACGGTAAGC	AGCCGTTACAAACAAAAACTTCCCTGGCAA
X b	TTGCCAGGGAAGTTTTTGTTTGTAACGGCT	ACTTTTGCTCACTAGTTACGCCATATCTCTTT
XI a	GATCTAGACTCGAGGGTACCATGAATTCCTCTCAAG	CTCAGAGCTTCTAGAGTTATCTCTGAACTTATCGG
XI b	CCGATAAGTTCAGAGATAACTCTAGAAGCTCTGAG	AGCCGTTACAAACAAAAACTTCCCTGGCAA
XI c	TTGCCAGGGAAGTTTTTGTTTGTAACGGCT	ACTTTTGCTCACTAGTTACGCCATATCTCTTT
XII a	GATCTAGACTCGAGGGTACCATGAATTCCTCTCAAG	TAAACCAGAAGTTTCAATAATCTCCACATC
XII b	GATGTGGAGATTATTGAAACTTCTGGTTTA	ACTTTTGCTCACTAGTTACGCCATATCTCTTT
XIII a	GATCTAGACTCGAGGGTACCATGAATTCCTCTCAAG	CTGTGAATCTGGCGGATCTGGATTGATCCC
XIII b	GGGATCAATCCAGATCCGCCAGATTCACAG	AGACGGCTTATCTCGAGACTTTGGCCTATC
XIII c	GATAGGCCAAAGTCTCGAGATAAGCCGTCT	ACTTTTGCTCACTAGTTACGCCATATCTCTTT
XIV a	GATCTAGACTCGAGGGTACCATGAATTCCTCTCAAG	AAACCTATTTCTAAACCTGTTACCGGAAGA
XIV b	TCTTCCGGTAACAGGTTTAGAAATAGGTTT	ACTTTTGCTCACTAGTTACGCCATATCTCTTT
XV a	GATCTAGACTCGAGGGTACCATGAATTCCTCTCAAG	ATCTGGATTGATCCCCTTCGGATCAAAAACCG
XV b	TCGGTTTTTGATCCGAAGGGGATCAATCCAGAT	ACTTTTGCTCACTAGTTACGCCATATCTCTTT
PLSP1 a	GATCTAGACTCGAGGGTACCATGATGGTGATGATA	AAGTTCTTCTCCTTTACTTGAATCCTTAAT
PLSP1 b	ATTAAGGATTCAAGTAAAGGAGAAGAACTT	AGACTTCGTTGTCTCGAGATCTAGTTCATC
PLSP1 c	GATGAACTAGATCTCGAGACAACGAAGTCT	TACCCTCGACGGATCCCTATTGCTTATCCAC

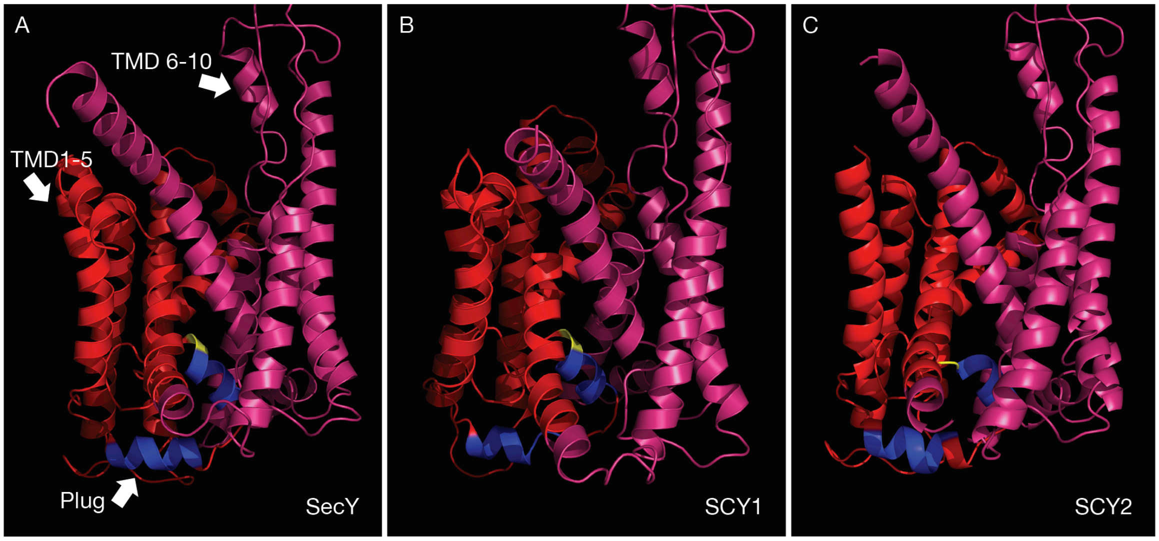

Figure S1. Molecular modeling of SecY and SCY proteins. *E.coli* SecY (PDB:3j45) was used as a template and the modeling was carried out at http://swissmodel.expasy.org/interactive. Despite the low degree of sequence identity, Arabidopsis SCY1 (B) and Arabidopsis SCY2 (C) can be fitted to the *E.coli* SecY (A) model, indicating they have similar structures. Each protein is made up of ten α-helixes where TMD1-5 (red) and TMD 6-10 (magenta) are arranged in a pseudo-symmetrical manner. A small α-helix forming the plug domain (blue) in the middle of the channel is also conserved in all the structures.

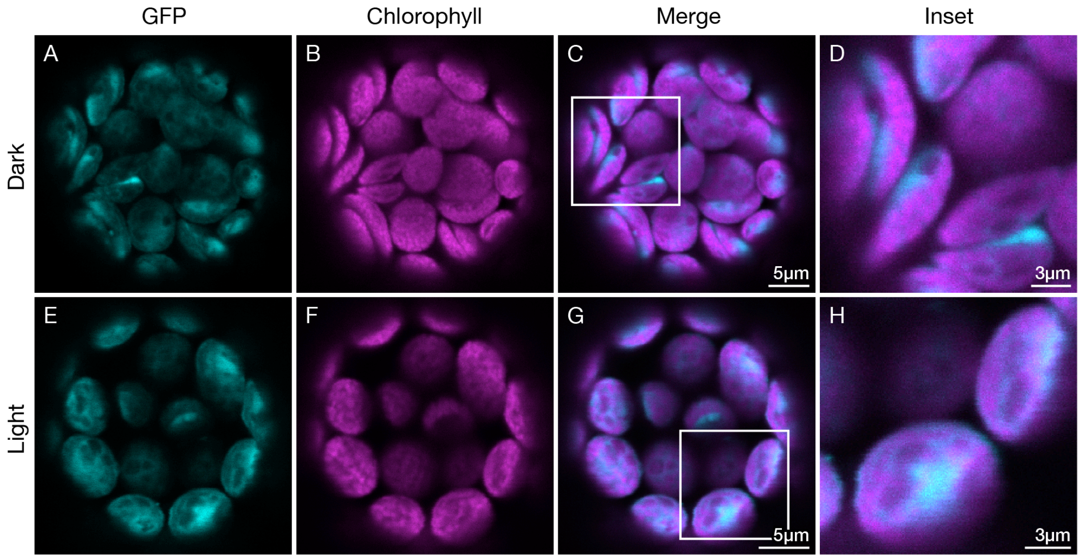

Figure S2. Targeting of small subunit of RUBISCO (SSU) in transfected protoplasts. Protoplasts from 3-week-old wildtype plants were transfected with a construct encoding SSU-GFP and incubated under either dark (A-D) or light (E-H) conditions for 12 h before imaging. GFP fluorescence (cyan), chlorophyll fluorescence (magenta), merged images, and higher magnification views (inset) of the boxed areas are shown. The SSU-GFP fusion construct contains SSU sequence from *Nicotiana tabacum* and was obtained from Prof. Enrico Schleiff (Department of Biosciences, Goethe University, Germany).

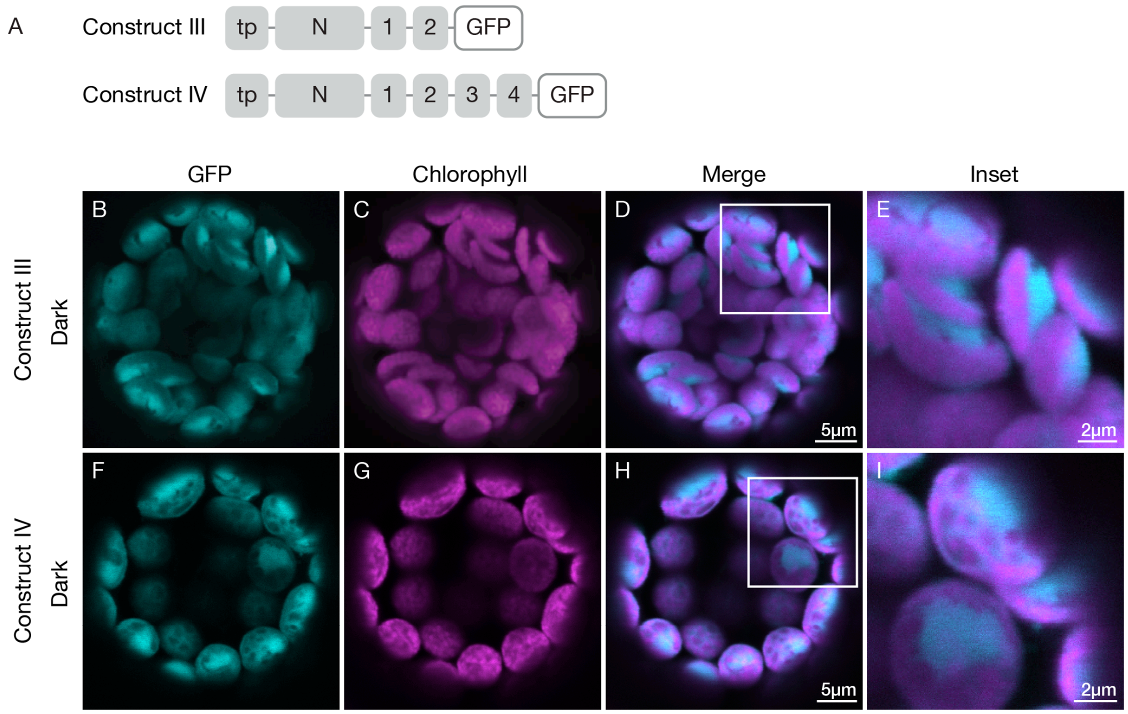

Figure S3. Localization of truncated SCY1 proteins in transfected protoplasts under dark conditions. (A) Structural representation of GFP constructs III and IV. Numbers indicate individual transmembrane domains. Protoplasts from 3-week-old wildtype (Ws) plants were transfected with construct III (B-E) or IV (F-I) and incubated under dark conditions for 12 h before imaging. GFP fluorescence (cyan), chlorophyll fluorescence (magenta), merged images, and higher magnification views (inset) of the boxed areas are shown. GFP: green fluorescent protein, N: N-terminal region, tp: transit peptide.

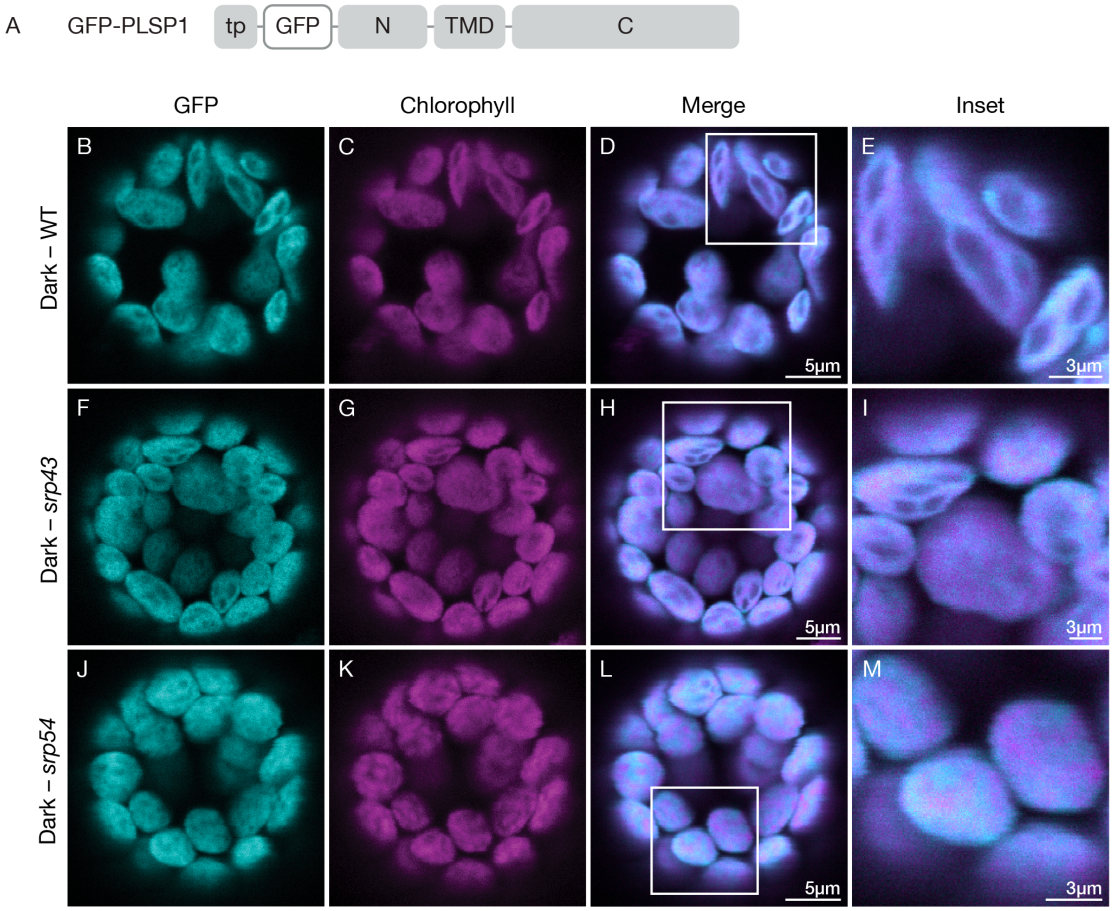

Figure S4: Targeting of PLSP1 (Plastidic Type 1 Signal Peptidase 1) to the thylakoids in wild type and *srp* mutants. To generate a fusion protein with GFP fused to the N-terminus of mature PLSP1 (AT3G24590), sequences encoding the PLSP1 transit peptide and mature PLSP1 protein were separately amplified from a citrine-PLSP1 clone (CRII gateway vector) obtained from Prof. Kentaro Inoue (UC-Davis) and introduced along with sequence encoding GFP between the KpnI and BamHI sites into the pML94 vector. (A) Structural representation of the GFP-PLSP1 construct. Protoplasts from 3-week-old wildtype (B-E), *srp43* mutant (F-I), or *srp54* mutant (J-M) plants were transfected with the GFP-PLSP1 construct and incubated for 12 h before imaging. GFP fluorescence (cyan), chlorophyll fluorescence (magenta), merged images, and higher magnification views (inset) of the boxed areas are shown. GFP: green fluorescent protein, N: N-terminal region, TMD: transmembrane domain, tp: transit peptide.
